# UAV Flight Altitude Measurement Based on AWA–AEIF Dual-Layer Information Fusion Algorithm

**DOI:** 10.3390/s26051552

**Published:** 2026-03-01

**Authors:** Qiqi Wu, Zhenwu He, Fan Zhang, Zhengrong Xiang, Xianming Xie, Lei Chen

**Affiliations:** 1 School of Automation, Guangxi University of Science and Technology, Liuzhou 545006, China; 100000327@gxust.edu.cn (Q.W.); 20230202015@stdmail.gxust.edu.cn (Z.H.); xxmxgm@163.com (X.X.); usrm6106@21cn.com (L.C.); 2 School of Physics and Information Engineering, Guangxi Science & Technology Normal University, Laibin 546199, China; 3 School of Mechanical Engineering and Electronic Information, China University of Geosciences, Wuhan 430074, China

**Keywords:** unmanned aerial vehicle (UAV), flight altitude measurement, barometric altimetry, multi-sensor data fusion, real-time differential measurement

## Abstract

**Highlights:**

**What are the main findings?**

- Architecture for reducing atmospheric effects in barometric altimetry.
- Method for reducing errors in barometric altimetry.

**What is the implication of the main finding?**

- The architecture is composed of a barometric reference station and a mobile station, enabling real-time differential pressure measurement.
- The method involves simultaneous barometric measurements using multiple barometer sets and data fusion with the AWA–AEIF algorithm.

**Abstract:**

Accurate altitude estimation is critical for unmanned aerial vehicles (UAVs), yet barometric measurements are susceptible to atmospheric drift and dynamic disturbances. To address these limitations, this paper proposes a dual-layer, real-time differential barometric altimetry framework that integrates a ground reference station with an onboard fusion scheme based on Adaptive Weighted Averaging (AWA) and an Adaptive Extended Information Filter (AEIF). The ground reference station suppresses low-frequency atmospheric variations, while the onboard AEIF incorporates a physical pressure–height model and adaptive noise estimation to maintain a fast dynamic response. The proposed method is validated through numerical simulations, hardware-in-the-loop (HIL) experiments, and real flight tests. In a two-hour outdoor flight test, compared with barometric systems operating without a reference station, the proposed approach reduces the altitude RMSE from 4.05 m to 0.31 m, achieving an approximately order-of-magnitude improvement in representative scenarios and demonstrating decimeter-level altitude measurement accuracy.

## 1. Introduction

Flight altitude is a critical parameter for unmanned aerial vehicles (UAVs) and a prerequisite for achieving autonomous flight in complex environments. Sensors commonly used for altitude measurement include accelerometers, barometers, Light Detection and Ranging (LiDAR), the Global Positioning System (GPS), Real-Time Kinematic (RTK) positioning, and vision systems. However, each sensor has inherent limitations. Accelerometers provide high-resolution vertical acceleration, but integration drift can cause divergence in altitude estimation [1,2]. Barometers estimate altitude from temperature and pressure changes, but they are highly sensitive to environmental temperature–pressure fluctuations [3,4]. LiDAR measures height using time-of-flight or phase difference, yet its performance depends on the reflective properties of the target surface [5,6]. Although GPS can provide altitude information, its vertical accuracy is substantially lower than its horizontal accuracy, and errors can exceed 10 m under cloudy conditions; therefore, GPS altitude is typically assigned limited weight in altitude fusion [7,8]. RTK improves accuracy via base-station corrections but increases equipment cost and deployment complexity [9,10]. Vision-based methods estimate altitude using feature points or stereo disparity, but they are constrained by lighting and texture conditions [11,12]. Consequently, due to physical and operational constraints, no single sensor can consistently achieve accurate UAV altitude measurement. Frequency-modulated continuous-wave (FMCW) radar can provide accurate range-to-ground sensing and is less affected by slow atmospheric pressure drift; however, it increases payload, power consumption, and cost, and its accuracy may degrade due to reflectivity, incidence angle, multipath, and cluttered or complex terrain [13,14].

From a physical perspective, barometric altitude estimation relies on the well-established relationship between atmospheric pressure and altitude. Under standard atmospheric conditions, pressure decreases monotonically with increasing altitude according to the barometric formula. Therefore, vertical motion of a UAV induces pressure variations that can be converted into altitude changes. In essence, the physical process follows a causal chain: vertical acceleration leads to changes in velocity, velocity changes lead to altitude variation, and altitude variation causes changes in pressure and temperature. Barometric altimetry thus provides a compact and lightweight solution; however, its accuracy is affected by atmospheric instability and sensor noise, motivating the use of differential sensing and adaptive fusion strategies.

To improve UAV altitude estimation, multi-sensor information fusion has become a major research focus, and existing methods differ substantially in absolute accuracy, environmental adaptability, and dynamic performance. Zhang et al. [15] employed an Extended Kalman Filter (EKF) to integrate GPS, MEMS-IMU, and barometric data, achieving sub-meter accuracy in hilly terrain; however, barometer temperature drift highlighted calibration challenges for low-cost sensors under dynamic atmospheric conditions. For complex indoor environments, Pritzl et al. [16] combined 3D LiDAR and IMU data using an adaptive multi-rate EKF, achieving an altitude RMSE of 0.74–1.84 m; nevertheless, LiDAR degradation in geometrically symmetric scenes indicates limited robustness of certain heterogeneous fusion approaches. Vision-aided methods, such as the hybrid fisheye–perspective camera system proposed by Eynard et al. [12], reported errors below 3.14% over unobstructed ground, but this increased to 8.82% in textureless environments, revealing a bottleneck in environmental adaptability. Huang et al. [17] proposed a multi-layer spatiotemporal fusion algorithm and achieved an RMSE below 0.1 m, but further optimization is required for sensor synchronization and robustness in extreme environments. In landing scenarios, Mu et al. [18] achieved a static accuracy of 0.043 m using an improved YOLOv4 and vision–IMU fusion, although hardware resource constraints may limit adaptability during high-speed maneuvers. Bassolillo et al. [19] used a multi-rate EKF to fuse IMU data, five Time-of-Flight (ToF) sensors, and an optical-flow camera, achieving an MAE of 0.0012 m; however, sensor noise and multi-rate synchronization led to error fluctuations at high velocities, reflecting challenges in real-time heterogeneous integration. Recent studies have explored AI-based altitude estimation to learn complex patterns in unstructured environments [20,21]. However, data-driven methods often require large training datasets and may lack interpretability and real-time robustness. In contrast, this work adopts a physics-informed, model-based fusion framework for reliable real-time UAV altimetry.

Within multi-source information fusion for altimetry, barometers are widely used due to their compactness and direct pressure-based altitude observability; however, they are affected by environmental disturbances, resulting in low-frequency drift and high-frequency noise, particularly in long-endurance UAV operations. To mitigate these drawbacks, recent research can be broadly summarized along three complementary directions. First, barometric measurements are fused with other sensing modalities to improve observability and overall accuracy, such as loosely coupled Kalman-filter-based fusion with map-matching or GNSS/INS aiding, hybrid schemes combining inertial sensing and learning-based motion patterns, and barometer-assisted indoor ranging systems [22,23,24,25,26,27]. A representative outcome is that vertical errors can be reduced from the multi-meter level typical of standalone GNSS/barometric solutions to sub-meter accuracy under favorable conditions by leveraging complementary observability from aided sensors. Second, the measurement architecture is enhanced to suppress common environmental disturbances, notably through dual-barometer configurations and reference–mobile station designs, including improved multi-barometer arrangements and atmospheric correction using auxiliary measurements such as temperature and humidity [28,29,30,31,32]. A representative result is that dual-barometer architectures can reduce altitude error by approximately 20–30% compared with conventional absolute barometric methods, particularly against slow atmospheric variations. Third, increasing attention has been paid to strengthening adaptive filtering in navigation by adopting adaptive covariance modeling and federated/variational Bayesian adaptive fusion, aiming to maintain stable performance under nonstationary noise and dynamic disturbances—conditions that are particularly challenging for the barometric vertical channel in real flight environments [33,34,35,36]. A representative advantage is that adaptive covariance modeling can maintain consistent estimation quality across changing disturbance levels without manual retuning, thereby improving reliability under time-varying conditions.

These advances collectively motivate a system-level integrated solution. Rather than optimizing only one aspect (sensor fusion, sensing architecture, or adaptive estimation), our approach combines sensor-aided fusion for observability, architecture-level differential sensing for drift suppression, and adaptive estimation for nonstationary disturbances to achieve simultaneous long-term drift suppression and strong dynamic responsiveness in real-time UAV altimetry. Based on the advantages of barometric altimetry, this paper proposes a real-time differential barometric altimetry method based on a dual-layer information fusion algorithm that integrates differential pressure measurement with multi-sensor fusion. The proposed method adopts a collaborative mode between a ground-end temperature–pressure reference station and a UAV-end temperature–pressure mobile station. By employing AWA and AEIF to fuse sensor data at the ground and UAV ends, respectively, measurement accuracy and robustness are improved. The target altitude measurement accuracy is defined as decimeter-level for indoor measurements and meter-level for outdoor measurements.

The novelty of this work lies in both the system-level sensing architecture and the filter modeling tailored for real-time UAV altimetry. The main contributions are summarized as follows:

1. A dual-layer sensing architecture is proposed, which tightly couples a ground-based reference station with a UAV-end estimator through real-time differential barometric measurements, enabling long-term drift suppression while preserving dynamic altitude response.
2. A filtering model is developed by integrating the Extended Information Filter (EIF) with the physical barometric altitude formulation, achieving an effective balance between multi-sensor fusion accuracy and fast dynamic response. This formulation is particularly suitable for real-time UAV applications with limited onboard computational resources.
3. An adaptive noise modeling and robust fusion strategy is incorporated into the UAV-end estimation framework, enabling the system to handle time-varying sensor noise and environmental disturbances. This joint design enhances fusion robustness under dynamic flight conditions, as validated by simulations, HIL tests, and real flight experiments.

## 2. Methods for UAV Altitude Measurement

### 2.1. Principle of Barometric Altimetry

Based on the principle that atmospheric temperature and pressure decrease as altitude increases, given the temperature and pressure at both the reference point and the measurement point, the altitude difference between them (denoted as *h*) can be calculated using the following pressure–height formula [37]:

(1) $$h=18410\left(1+\frac{\bar{T}}{273.15}\right)log\frac{P_{R}}{P_{M}}$$

where *P_R_* and *P_M_* are the atmospheric pressures at the reference point and the measurement point, respectively; $\bar{T}$ is the average temperature between the two points, which can be obtained from the arithmetic mean of the temperature at the reference point (denoted as *T_R_*) and the temperature at the measurement point (denoted as *T_M_*):

(2) $${\bar{T}}_{m}=\left(T_{R}+T_{M}\right)/2$$

To facilitate analysis and calculation, the barometric formula is often expressed in its natural logarithm form. For this purpose, a function *M*(.) is introduced, and the formula is rewritten as follows:

(3) $$h=M\left(T_{R},P_{R},T_{M},P_{M}\right)=7995.4\left(1+\frac{T_{R}+T_{M}}{546.3}\right)\ln\frac{P_{R}}{P_{M}}$$

Practical measurements indicate that when the horizontal distance between the reference point and the measurement point is less than 50 km, the error of Equation (3) is approximately 1–2 m [37].

### 2.2. Pseudo-Differential Barometric Altimetry Method

The pseudo-differential barometric altimetry method is a technique widely applied in UAVs, which can be described as follows: the ground-end temperature and pressure values (*T_R_*, *P_R_*) measured prior to takeoff are used as the reference values, which are then substituted into the pressure–height formula along with the real-time UAV-end temperature and pressure values (*T_M_*, *P_M_*) measured during flight to calculate the flight altitude. This method is termed “pseudo-differential” because the temperature–pressure difference used in the formula is not based on synchronized observations at the same timestamp; the reference temperature/pressure at the ground end is not constant during the flight.

Variations in ground atmospheric parameters during flight are the primary source of error. Let the changes in ground temperature and pressure be denoted as *T_e_* and *P_e_*, respectively. The resulting error in the calculated altitude is denoted as *h_e_*, which can be expressed quantitatively by the following formula:

(4) $$h_{e}=M\left(T_{R},P_{R},T_{M},P_{M}\right)-M\left(T_{R}+T_{e},P_{R}+P_{e},T_{M},P_{M}\right)$$

To analyze the independent effects of *T_e_* and *P_e_* on *h_e_*, a sensitivity analysis of *h_e_* is required. This involves calculating the first-order partial derivatives with respect to *T_e_* and *P_e_* to evaluate their respective contributions. The expressions for the partial derivatives are as follows:

(5) $$\left\{\begin{array}{c} \frac{\partial h_{e}}{\partial T_{e}}=-14.64\ln\frac{P_{R}+P_{e}}{P_{M}}\\ \frac{\partial h_{e}}{\partial P_{e}}=-7995.4\left(1+\frac{T_{R}+T_{M}+T_{e}}{546.3}\right)\frac{1}{P_{R}+P_{e}} \end{array}\right.$$

**Figure 1:**
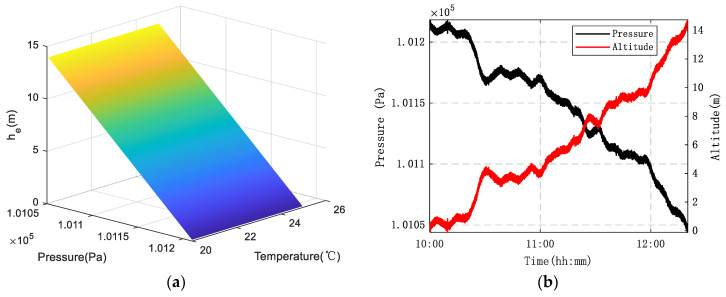
Altitude measurement errors caused by the meteorological environment: (**a**) Theoretical values; (**b**) Measured values.

A visualization was performed based on Equation (5). Simulation results (Figure 1a) demonstrate that a scenario involving a 160 Pa decrease in ground pressure and a 5 °C increase in temperature can induce an error of approximately 14 m. Field measurements were conducted between 10:00 and 12:30, and the test results (Figure 1b) are basically consistent with these findings. Therefore, it is evident that dynamic variations in temperature/pressure at the reference point significantly increase measurement errors.

### 2.3. Real-Time Differential Barometric Altimetry Method

Unlike the pseudo-differential method, the real-time differential barometric altimetry method synchronizes the measurements of the ground-end *T_R_* and *P_R_* and the UAV-end *T_M_* and *P_M_* in real time. These four parameters are then substituted into Equation (3) to calculate the altitude directly. Since the reference values are strictly synchronized with the measured values, errors induced by time-varying ground atmospheric conditions can theoretically be eliminated. Consequently, the measurement precision at this stage primarily depends on the inherent measurement errors of the barometers and thermometers themselves.

**Figure 2:**
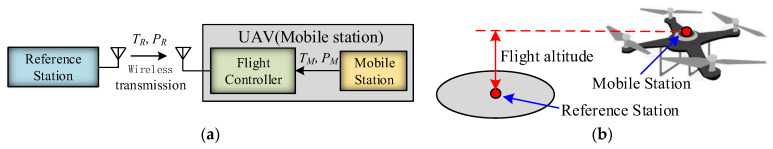
Real-time differential barometric altimetry system: (**a**) Structural diagram; (**b**) Measurement scenario.

The hardware architecture of the real-time differential barometric altimetry system is illustrated in Figure 2, consisting of two primary components: the ground-end reference station and the UAV-end mobile station. The reference station is responsible for the measurement of *T_R_* and *P_R_* and transmits this data to the UAV in real time via a wireless data link. The mobile station, mounted on the UAV, is utilized for the synchronized measurement of *T_M_* and *P_M_*. The flight controller receives these parameters from both stations and fuses the data to compute the flight altitude in real time.

To quantify the uncertainty of the real-time differential barometric altimetry results, the standard deviation of the altitude measurement, denoted as σ*_h_*, is employed as the evaluation metric. Provided that the standard deviations *σ_mi_* of all input quantities *m_i_* (namely, *T_R_*, *P_R_*, *T_M_*, and *P_M_*) are known, σ*_h_* can be calculated according to the law of error propagation; the derivation process of the calculation formula is provided in Appendix A.

**Figure 3:**
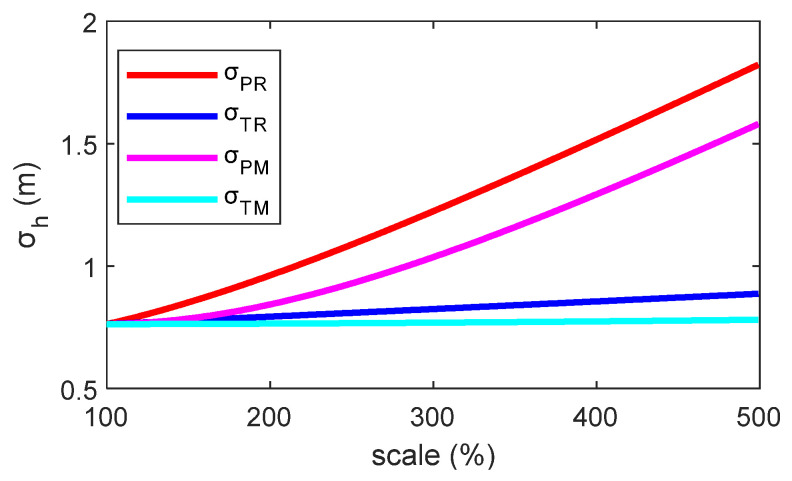
Impact of input parameter uncertainties on altitude estimation variance.

To analyze the influence of individual measurement errors on the accuracy of altitude determination, a parametric sensitivity analysis was conducted using Equation (A7). The benchmark parameters were set as follows: *P_R_* = 101325 Pa, *T_R_* = 20 °C, *P_M_* = 100150 Pa, and *T_M_* = 19.35 °C, corresponding to a theoretical altitude *h* = 100 m. The initial standard deviations for the input quantities were set as σ*_PR_* = 4 Pa, σ*_TR_* = 0.2 °C, σ*_PM_* = 4 Pa, and σ*_TM_* = 0.2 °C (these parameters were referenced from physical sensors). Subsequently, using the univariate analysis method, the standard deviation of each parameter was varied sequentially while keeping the others constant. The resulting trend in the combined standard deviation of the calculated altitude, σ*_h_*, is shown in Figure 3. The results indicate a positive correlation between σ*_h_* and the standard deviation of each input quantity.

### 2.4. Other UAV Altitude Measurement Methods

The GPS and accelerometers are indispensable sensors in UAV flight controllers. Although the GPS can measure altitude, its vertical accuracy is relatively low (typically with errors exceeding 10 m), which limits its utility in high-precision measurement applications. In contrast, its velocity measurement is relatively precise, with a standard deviation that is typically within 0.2 m over short periods. Therefore, GPS velocity information is often utilized, and altitude change is estimated through integration. Denoting the vertical velocity as *v*, the altitude change (denoted as Δ*h_gps_*) obtained by integration within the time interval (denoted as Δ*T*) is as follows:

(6) $$\Delta h_{gps}=\int_{0}^{\Delta T}vdt$$

Accelerometers can also be utilized to measure the relative altitude of UAVs, based on the principle of double integration of the vertical acceleration (denoted as *a_z_*). Specifically, the vertical motion acceleration of a UAV (denoted as *a*) is calculated by combining the accelerometer measurements with the UAV’s attitude angles. Driven by the vertical movement acceleration, the specific altitude change generated by the UAV within the time interval Δ*T* is denoted as Δ*h_acc_*, which can be calculated using the following formula:

(7) $$\Delta h_{acc}=\int_{0}^{\Delta T}dt\int_{0}^{\Delta T}adt$$

The measurement error of acceleration will lead to an error in the altitude calculation. This propagation relationship can be expressed by the following formula:

(8) $$h_{err}=\Delta h_{acc}-\Delta h_{true}=\int_{0}^{\Delta T}dt\int_{0}^{\Delta T}\left(a-a_{ture}\right)dt=\int_{0}^{\Delta T}dt\int_{0}^{\Delta T}a_{err}dt$$

where Δ*h_ture_* and *h_err_* represent the true altitude variation and the altitude error, respectively; *a_ture_* and *a_err_* denote the true acceleration and the acceleration error, respectively. Equation (8) indicates that *h_err_* accumulates rapidly over time (i.e., it diverges). Therefore, the method of measuring altitude using accelerometers is typically suitable only for short-term measurement scenarios.

## 3. Data Fusion Algorithm

Based on the preceding analysis, the GPS, barometers, and accelerometers possess distinct advantages in measuring absolute and relative altitudes. Accordingly, a novel UAV altitude measurement algorithm is proposed in this paper, aiming to leverage the strengths of these sensors.

### 3.1. Algorithm Structure

According to error propagation theory, the total uncertainty of altitude measurement is related to the individual uncertainty components of each sensor. Therefore, one direct approach to reduce measurement error is by employing multi-sensor synchronized measurement followed by averaging to decrease the measurement standard deviation. Assuming that *n* sensors with identical precision are used for synchronized independent measurements, each with a standard deviation of *σ*, the standard deviation of the arithmetic mean of these *n* measurements (denoted as *σ*_1_) is calculated as follows:

(9) $$\sigma_{1}=\sqrt{\frac{1}{n^{2}}\sum_{i=1}^{n}\sigma_{i}^{2}}=\sqrt{\frac{\sigma^{2}}{n}}=\frac{\sigma }{\sqrt{n}}$$

As shown in Equation (9), the standard deviation of the measurement result decreases as the number of sensors increases. Therefore, this method is theoretically validated as feasible.

**Figure 4:**
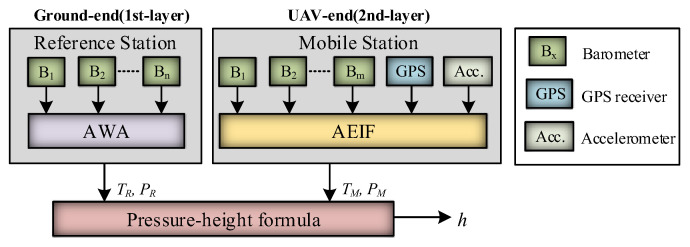
Schematic of the dual-layer AWA–AEIF information fusion framework.

It is noteworthy that the temperature–pressure observation environments and variation patterns of the data received from the ground-end reference station (static) and the UAV-end mobile station (dynamic) are distinct, necessitating different data fusion strategies. A computationally efficient AWA algorithm was designed to address the strong stationarity of the ground-end temperature–pressure data, whereas an AEIF algorithm was developed to handle the highly dynamic characteristics of the UAV-end temperature–pressure data. Meanwhile, the high-precision relative altitude variations provided by the GPS and accelerometers serve as the state prediction inputs for the AEIF algorithm. Based on the above analysis, a dual-layer information fusion algorithm, referred to as the AWA–AEIF algorithm, was designed. This framework is not based on a single-platform pre-fusion followed by filtering; rather, it is a two-node reference–UAV architecture in which a ground reference station (first layer) and an onboard estimator (second layer) are tightly coupled through synchronized real-time differential measurements. The algorithm structure is illustrated in Figure 4.

The algorithm procedure is as follows: First, the measurement values from *n* barometers at the reference station (equipped with both pressure and temperature sensing capabilities) are fused via the AWA algorithm to output the optimal estimates of ground-end *T_R_* and *P_M_*. Subsequently, the measurement values from *m* barometers, GPS, and accelerometers at the mobile station are fused via the AEIF algorithm to output the optimal estimates of *T_M_* and *P_M_*. Finally, these four parameters are substituted into the pressure–height formula to calculate the flight altitude.

### 3.2. Estimation of Ground-End Temperature and Pressure

Although multi-sensor fusion can effectively reduce the measurement standard deviation, the actual standard deviation of each individual sensor is often inconsistent and time-varying. Therefore, applying a simple arithmetic mean is not the optimal fusion strategy. To this end, fusion weights must be dynamically allocated based on the real-time estimated standard deviation of each sensor. An algorithm known as Adaptive Weighted Averaging (AWA) was designed. Given that the time-varying characteristics of ground-end temperature and pressure are typically slow, the AWA algorithm is well-suited for this fusion task.

Consider the synchronized measurement of a specific parameter using *n* sensors. The discrete time step is denoted as *t*, and the raw measurement from the *i*-th sensor is denoted as *m_i_*. Its mean and standard deviation, obtained through recursive estimation, are denoted as ${\bar{m}}_{i}$ and *σ_i_*, respectively. The weight assigned to each sensor in the fusion process is denoted as *w_i_*, the weighted average result is *m_t_*, and the final fusion result is *fm*. In this algorithm, the recursive updates of the mean and standard deviation incorporate a forgetting factor (denoted as *b*) and a smoothing time constant (denoted as *s*) to enhance the influence of recent data. The specific execution steps of the AWA algorithm are as follows:

Step 1: Mean update:

(10) $${\bar{m}}_{i,t}=b{\bar{m}}_{i,t-1}+\left(1-b\right)m_{i,t}$$

Step 2: Measurement residual calculation:

(11) $$e_{i,t}=m_{i,t}-{\bar{m}}_{i,t}$$

Step 3: Standard deviation update:

(12) $$\sigma_{i,t}=\sqrt{b\sigma_{i,t-1}^{2}+\left(1-b\right)e_{i,t}^{2}}$$

Step 4: Weight calculation:

(13) $$w_{i,t}=\frac{1}{\sigma_{i,t}^{2}}$$

Step 5: Weighted fusion execution:

(14) $$m_{t}=\frac{\sum_{i=0}^{n}m_{i,t}\cdot w_{i,t}}{\sum_{i=0}^{n}w_{i,t}}$$

Step 6: Smoothing of fused values:

(15) $${fm}_{t}=sfm_{t-1}+(1-s)m_{t}$$

In the algorithm, the forgetting factor is selected according to the temporal characteristics of the measured signals. In practice, values in the range of 0.95–0.99 provide a good trade-off between estimation smoothness and responsiveness. Specifically, for the ground reference station, where temperature and pressure variations are slow and dominated by low-frequency atmospheric trends, a larger forgetting factor is adopted to enhance noise suppression and estimation stability. In contrast, for the UAV-end measurements, which exhibit rapid variations due to altitude changes and dynamic flight maneuvers, a slightly smaller forgetting factor is employed to maintain sufficient responsiveness to fast signal changes. Within these ranges, the algorithm performance is not sensitive to small variations in the forgetting factor.

### 3.3. Estimation of UAV-End Pressure and Temperature

#### 3.3.1. Measurement System Analysis

The temperature/pressure at the UAV end changes rapidly with flight altitude and motion status, exhibiting significant dynamic characteristics. In this context, the AWA algorithm—originally designed for slowly changing signals—struggles to provide accurate estimates for such dynamic signals due to its inherent latency. Consequently, it is essential to employ estimation algorithms capable of effectively fusing dynamic measurements, such as the Kalman Filter (KF). The KF algorithm is based on a “predict–correct” framework: it first predicts the state using a system model and then refines the prediction with sensor measurements, thereby achieving real-time, optimal estimation of system states (e.g., dynamic temperature–pressure estimation).

Consider the following discrete-time system:

(16) $$\left\{\begin{array}{l} x_{t}=f(x_{t-1},w_{x,t})+g\left(u_{t},w_{u,t}\right),w_{x,t}\sim N(0,Q_{x,t}),w_{u,t}\sim N(0,Q_{u,t})\\ z_{t}=h(x_{t},n_{t}),\;n_{t}\sim N(0,R_{t}) \end{array}\right.$$

where *f*(·), *g*(·), and *h*(·) denote the state transition function, control input function, and observation function, respectively; ***x***, ***u***, and ***z*** represent the system state vector, control input vector, and measurement vector, respectively; and ***w_x_***, ***w_u_***, and ***n*** signify the process noise, control noise, and measurement noise, respectively, with their corresponding covariance matrices being ***Q_x_***, ***Q_u_***, and ***R***. Specifically regarding the UAV altitude measurement, during the prediction stage of the KF algorithm, the flight altitude can be recursively estimated based on the kinematic laws of the UAV in the vertical direction (described by functions *f* and *g*), which, in turn, predicts the UAV-end temperature and pressure. In the correction stage, the predicted states are refined according to the real-time observations (***z***) from the barometers.

#### 3.3.2. System State Prediction

To construct a state-space model suitable for the Kalman Filter, it is necessary to precisely define the state vector and the control vector. In this paper, the UAV-end temperature and pressure (*P_M_*, *T_M_*) and the vertical velocity (*v*) are defined as the state vector (***x***), while the vertical acceleration (*a*) is defined as the control input (***u***), expressed as follows:

$$x={\left[\begin{array}{ccc} P_{M} & T_{M} & v \end{array}\right]}^{T},u=\left[a\right]$$

**Figure 5:**
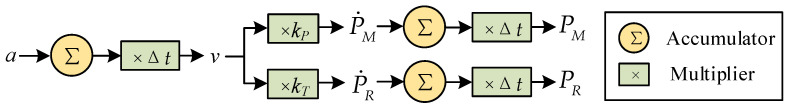
Physical evolution and transition logic of the UAV state variables.

The dynamic transition relationships between the state variables are illustrated in Figure 5. The physical interpretation of the UAV state variables and their transition dynamics is as follows: During flight, the vertical acceleration of the UAV first leads to variations in vertical velocity, and the change in velocity subsequently results in a change in altitude. Since atmospheric pressure and temperature are physically related to altitude, variations in altitude directly induce corresponding changes in pressure and temperature. From a physical perspective, the state transition process therefore follows a causal chain of “acceleration → velocity → altitude → pressure and temperature”. In other words, the UAV acceleration indirectly drives the evolution of pressure and temperature through its kinematic effect on altitude, which constitutes the physical basis of the adopted state transition dynamics.

Their physical essence can be described by a set of discretized differential equations, where *v* is the integral of *a* with respect to time; the rate of change of pressure (denoted as ${\dot{P}}_{M}$) is linearly related to *v* (with a proportional coefficient *k_P_*); and *P_M_* is the integral of its rate of change ${\dot{P}}_{M}$. The variation process of *T_M_* is similar to that of *P_M_*, following a specific vertical temperature gradient model. The operational cycle of the algorithm is denoted as Δ*t*.

Although the physical conversion process is continuous, it needs to be discretized for application in filtering algorithms. Based on the transition relationships depicted in Figure 5, the discrete-time state evolution of the system can be preliminarily expressed in the following recursive form:

(17) $$v=\sum \Delta ta,P_{M}=\sum \Delta tk_{P}v,T_{M}=\sum \Delta tk_{T}v$$

where *k_P_* and *k_T_* are the key proportional coefficients. *k_P_* characterizes the relationship between the vertical velocity and the rate of pressure change, and can be defined as follows:

(18) $$k_{p}=\frac{{\dot{P}}_{M}}{v}=\frac{\left(\Delta P_{M}/\Delta t\right)}{\left(\Delta h/\Delta t\right)}=\frac{\Delta P_{M}}{\Delta h}$$

To solve for *k_P_*, an analytical relationship between *P_M_* and *h* must be established. By inverting the pressure–height formula (Equation (3)), the following expression is obtained:

(19) $$P_{M}=P_{R}\cdot e^{\frac{-h}{14.635\left(546.3+T_{R}+T_{M}\right)}}$$

Substituting Equation (19) into Equation (18) and approximating the differences with partial differentials, the analytical expression for *k_P_* can be derived as follows:

(20) $$k_{P}=\frac{\Delta P_{M}}{\Delta h}\approx \frac{\partial P_{M}}{\partial h}=\frac{-P_{M}}{14.635\left(546.3+T_{R}+T_{M}\right)}$$

Similarly, *k_T_* characterizes the relationship between the vertical velocity and the rate of temperature change. According to the standard atmospheric model, the temperature decreases by approximately 6.5 °C for every 1 km increase in altitude, i.e., the vertical temperature gradient is about −0.0065 °C/m [37]. Thus, the coefficient is given by the following equation:

(21) $$k_{T}=\frac{{\dot{T}}_{M}}{v}=\frac{\left(\Delta T_{M}/\Delta t\right)}{\left(\Delta h/\Delta t\right)}=\frac{\Delta T_{M}}{\Delta h}=-0.0065\;{}^{\circ}C/m$$

Based on the dynamic relationships of the state variables (Figure 5) and the coefficients *k_P_* and *k_T_*, the continuous differential equations are discretized (with a sampling period of Δ*t*) to obtain the complete state transition equation for the KF prediction step. This equation maps the previous state ***x****_t−_*_1_ and the control input ***u****_t_* to the predicted current state ***x****_t_*:

(22) $$x_{t}=f(x_{t-1},w_{x,t})+g\left(u_{t},w_{u,t}\right)=\left[\begin{array}{c} P_{M,t-1}+k_{P}v_{t-1}\Delta t+k_{P}a_{t}\Delta t^{2}/2\\ T_{M,t-1}+k_{T}v_{t-1}\Delta t+k_{T}a_{t}\Delta t^{2}/2\\ v_{t-1}+a_{t}\Delta t \end{array}\right]+w_{t}$$

where ***w_t_*** represents the total process noise.

#### 3.3.3. System State Observations

The observations of the system are provided by the barometers and GPS. Specifically, the temperature and pressure values synchronously measured by the barometers are denoted as *T_baro_* and *P_baro_*, which correspond to *T_M_* and *P_M_* in the system states, respectively. The vertical velocity measurement provided by the GPS (denoted as *v_gps_*) corresponds to *v* in the state vector. Consequently, these three direct observations constitute the system’s observation vector ***z***, expressed as

$$z={\left[\begin{array}{ccc} P_{baro} & T_{baro} & v_{gps} \end{array}\right]}^{T}$$

The observation equation is as follows:

(23) $$z_{t}=h\left(x_{t},n_{t}\right)=x_{t}+n_{t}\Leftrightarrow \left\{\begin{array}{c} P_{baro,t}=P_{M,t}+n_{P,t}\\ T_{baro,t}=T_{M,t}+n_{T,t}\\ v_{gps,t}=v_{t}+n_{v,t} \end{array}\right.$$

According to Equation (23), the observation equation is of a linear form. Consequently, the system’s observation model can be directly represented linearly by the observation matrix (denoted as ***H***), as shown below:

(24) $$z_{t}=Hx_{t}=\left[\begin{array}{ccc} 1 & 0 & 0\\ 0 & 1 & 0\\ 0 & 0 & 1 \end{array}\right]x_{t}+n_{t}$$

#### 3.3.4. Multi-Sensor Fusion Algorithm

To enhance the reliability and measurement accuracy of UAV flight control, multiple sets of homogeneous sensors (e.g., multiple barometers and GPS units) are often deployed within the system. To fully utilize the redundant or complementary observation information from these sensors, effective data fusion algorithms are required. Within the Kalman Filter framework, an efficient and straightforward implementation is the Centralized Dimension-Augmented Fusion Algorithm.

The principle of this algorithm is as follows: For a discrete-time system comprising *m* sensor subsystems, if the observation equation is linear, the observation matrix can be expressed in the following form:

(25) $$z_{t,i}=H_{i}x_{t}+n_{t,i},i=1,2,\cdots ,m$$

where ***z****_i_*, ***H****_i_*, and ***n_i_*** represent the observation vector, observation matrix, and measurement noise of the *i*-th sensor subsystem, respectively. The covariance matrix corresponding to ***n****_i_* is denoted as ***R****_i_*. To achieve centralized optimal fusion of measurement data from all sensors, the observation models of each subsystem need to be concatenated and integrated at the vector and matrix dimension levels. This method is referred to as dimension-augmented fusion. Specifically, the dimension augmentation is performed on ***z****_i_*, ***H****_i_*, ***n****_i_*, and ***R_i_*** as follows:

(26) $$\begin{array}{l} z={\left[z_{1}^{T},z_{2}^{T},\cdots ,z_{m}^{T}\right]}^{T},H={\left[H_{1}^{T},H_{2}^{T},\cdots ,H_{m}^{T}\right]}^{T},\\ n={\left[n_{1}^{T},n_{2}^{T},\cdots ,n_{m}^{T}\right]}^{T},R=diag\left(R_{1},R_{2},\cdots ,R_{m}\right) \end{array}$$

where ***z***, ***H***, ***n***, and ***R*** denote the observation vector, observation matrix, measurement noise, and noise covariance matrix after dimension augmentation, respectively. Then, the system observation equation after dimension augmentation can be expressed as follows:

(27) $$z_{t}=Hx_{t}+n_{t},n_{t}\sim N(0,R_{t})$$

#### 3.3.5. Dynamic Estimation of Sensor Standard Deviation

Similar to the ground-end measurements, the noise standard deviation (or variance) of each sensor in the UAV-end temperature–pressure measurement is difficult to calibrate precisely in advance and is time-varying, which tends to degrade the performance of multi-sensor fusion. To address this issue, this paper introduces the Sage–Husa adaptive filtering algorithm to dynamically estimate the noise standard deviation of each sensor online. Since the square of the standard deviation is the variance, this process is equivalent to the real-time estimation of the measurement noise covariance matrix (***R***). The calculation steps are as follows:

1. Calculate the dynamic weight coefficient (denoted as *d*): The weight coefficient for the current time step is calculated based on a predefined forgetting factor (*b*). This coefficient determines the contribution of the innovation to the covariance estimation, as expressed in the following formula:

(28) $$d_{t}=\frac{1-b}{1-b^{t+1}}$$

2. Calculate the innovation: The innovation is defined as the difference between the actual measurement and the predicted measurement. It contains information about both the model’s prediction error and the measurement noise. Its calculation formula is as follows:

(29) $$e_{t}=z_{t}-Hx_{t}$$

3. Update the noise covariance matrix: Utilize *d* and ***e*** to recursively update the estimate of the observation noise covariance matrix.

(30) $$R_{t}=\left(1-d_{t}\right)R_{t-1}+d_{t}\left(e_{t}^{2}-HPH^{T}\right)$$

where ***P*** represents the covariance matrix of the state vector. Through this recursive process, the algorithm can gradually converge to the true ***R*** over time. To ensure stable convergence of the proposed adaptive filtering framework, both preventive and protective measures are incorporated into the AEIF design.

As a preventive measure, since different sensing channels operate independently, the measurement noises from different sensors are assumed to be weakly correlated. To avoid spurious correlations introduced by online estimation, the measurement noise covariance matrix ***R*** is constrained to a diagonal form by eliminating its off-diagonal elements after each update, thereby ensuring that ***R*** remains positive definite.

As a protective measure, the initial sensor noise standard deviation (denoted as σ_0_) is used as a reference. If the estimated value of σ exceeds a predefined range (typically chosen to be between 0.2σ_0_ and 5σ_0_), the estimate is regarded as unreliable and is reset to its initial value. This mechanism effectively limits the influence of abnormal estimates and enhances robustness against transient disturbances.

#### 3.3.6. Dynamic Fusion Algorithm Design

To ensure that the designed fusion algorithm achieves the optimal balance of precision, real-time performance, and robustness, it is necessary to comprehensively weigh the following requirements:

- Computational efficiency requirement: As shown in Equation (26), in multi-sensor systems, the dimension of the observation matrix increases dramatically. This leads to a significant time cost associated with matrix inversion when calculating the Kalman gain in the Kalman Filter. In contrast, its dual form—the Information Filter (IF)—provides a significant speed advantage for multi-source data fusion as it avoids the explicit computation of the Kalman gain.
- Nonlinear processing requirement: The state transition Equation (22) presented in this study constitutes a nonlinear system due to the inclusion of nonlinear proportional terms, *k_p_*. Therefore, the standard IF cannot be directly applied, and it is necessary to employ its nonlinear extension, such as the Extended Information Filter (EIF).
- Adaptive capability requirement: The sensor noise covariance is difficult to precisely determine in practice. To enhance fusion accuracy and system robustness, the algorithm should possess the capability for online adaptive estimation of the noise statistics.

Based on the comprehensive requirements for computational efficiency, nonlinear processing, and adaptive capability, this study designs an algorithm named the Adaptive Extended Information Filter (AEIF) to serve as the fusion algorithm for the UAV-end temperature–pressure data. The AEIF is developed from the EIF [38], and its core mathematical framework is the IF form. While mathematically equivalent to the traditional KF, the IF uses the information vector (denoted as ***y***) and information matrix (denoted as ***Y***) to characterize the state and its uncertainty, as opposed to the conventional state vector (***x***) and covariance matrix (***P***). The reversible transformation relationship between the two representations is as follows:

(31) $$\begin{array}{l} Y_{t}=P_{t}^{-1}\\ y_{t}=Y_{t}x_{t}=P_{t}^{-1}x_{t} \end{array}$$

If the system described by Equation (16) is assumed to be linear, then the state transition and observation can be described by the state transition matrix (***F***), the control matrix (***G***), and the observation matrix (***H***), respectively. Under this premise, the execution process of the IF is similar to that of the KF, also consisting recursively of two stages: prediction and update. The formulas for the prediction stage are as follows:

(32) $$\begin{array}{l} Q_{t}=Q_{x,t}+G_{t}Q_{u,t}G_{t}^{T}\\ Y_{t|t-1}={\left[F_{t}Y_{t-1}^{-1}F_{t}^{T}+Q_{t}\right]}^{-1}\\ y_{t|t-1}=Y_{t|t-1}\left(F_{t}Y_{t-1}^{-1}y_{t-1}+G_{t}u_{t}\right) \end{array}$$

The formulas for the update stage are as follows:

(33) $$\begin{array}{l} Y_{t}=Y_{t|t-1}+H_{t}^{T}R_{t}^{-1}H_{t}\\ y_{t}=y_{t|t-1}+H_{t}^{T}R_{t}^{-1}z_{t} \end{array}$$

To apply the IF to barometric altimetry, it is necessary to analyze the prediction and update stages separately. In the prediction stage, due to their nonlinear characteristics, both the state transition function *f*(·) and the control input function *g*(·) must be linearized. A common approach is to calculate their Jacobian matrices, denoted as *J_f_* and *J_g_*, respectively. In the prediction equations, *J_f_* replaces the original linear-state transition matrix ***F***, while *J_g_* replaces the original control matrix ***G***. The specific calculation results for *J_f_* and *J_g_* are shown below:

(34) $$J_{f,t}=\frac{\partial f}{\partial x}=\left[\begin{array}{ccc} 1+(v_{z,t-1}\Delta t+a_{z}\Delta t^{2}/2)k_{P,t}/P_{M,t-1} & 0 & k_{P,t}\Delta t\\ 0 & 1 & k_{T}\Delta t\\ 0 & 0 & 1 \end{array}\right],J_{g,t}=\frac{\partial g}{\partial x}=\left[\begin{array}{c} k_{P,t}\Delta t^{2}/2\\ k_{T}\Delta t^{2}/2\\ \Delta t \end{array}\right]$$

During the update stage, owing to the linear characteristics of the observation equation, the observation matrix can be directly utilized for update calculation. To implement multi-sensor information fusion, a centralized dimension-augmented method is adopted: specifically, the system’s augmented observation vector (***z***), observation matrix (***H***), and noise covariance matrix (***R***) are constructed according to Equation (26). Furthermore, to handle the time-varying nature and uncertainty of sensor noise, the Sage–Husa adaptive filtering algorithm is embedded into the update process, where Equations (28)–(30) are executed synchronously.

**Figure 6:**
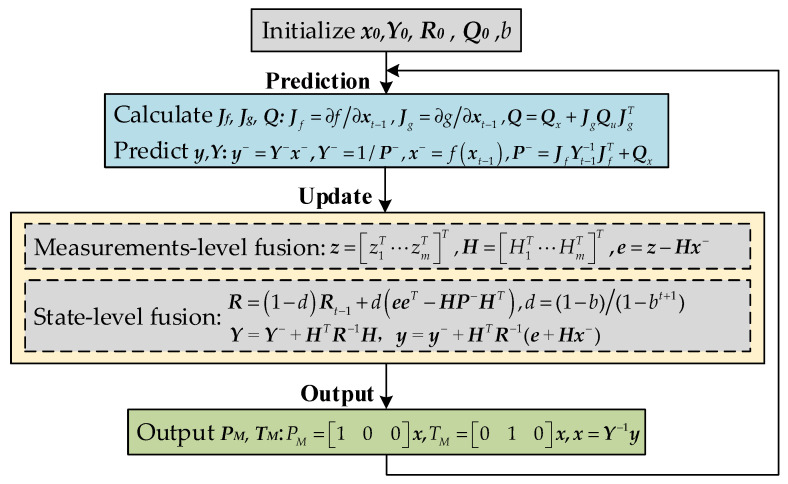
Computational flowchart of the AEIF algorithm.

Through the above analysis, the complete AEIF algorithm is developed by incorporating nonlinear processing and adaptive capabilities into the linear IF and by refining its front-end initialization and back-end output interfaces. The procedure is illustrated in Figure 6.

The algorithm procedure consists of four successive stages: initialization, prediction, update, and output. The specific details of each stage are described below.

Initialization phase: This stage involves the configuration of four key parameters: ***x***_0_ is initialized using sensor measurements; ***R***_0_ is initialized based on the sensor standard deviations; ***Q***_0_ is set according to the UAV’s kinematic characteristics. Consequently, the initial value of ***P***_0_ is calculated as follows:

(35) $${\bar{x}}_{0}=E(x_{0}),P_{0}=E[(x_{0}-{\bar{x}}_{0}){\left(x_{0}-{\bar{x}}_{0}\right)}^{T}]$$

Prediction phase: In this phase, *J_f_*, *J_g_*, and *Q* are computed, and ***y*** and ***Y*** are predicted.

Step 1: Calculate *J_f,t_*, *J_g,t_*, and *Q_t_*:

(36) $$J_{f,t}={\left.\frac{\partial f}{\partial x}\right|}_{x_{t-1}},J_{g,t}={\left.\frac{\partial g}{\partial x}\right|}_{x_{t-1}},Q_{t}=Q_{x,t}+J_{g,t}Q_{u,t}J_{g,t}^{T}$$

Step 2: Predict the system state, ***x****_t|t−_*_1_, and the covariance ***P****_t|t−_*_1_:

(37) $$x_{t|t-1}=f\left(x_{t-1}\right),P_{t|t-1}=J_{f,t}Y_{t-1}^{-1}J_{f,t}^{T}+Q_{x}$$

Step 3: Convert to information form:

(38) $$y_{t|t-1}=Y_{t|t-1}x_{t|t-1},Y_{t|t-1}=P_{t|t-1}^{-1}$$

Update phase: In this phase, measurement-level fusion and state-level fusion are implemented, where ***z*** is fused, ***R*** is estimated, and ***y*** and ***Y*** are updated.

Step 1: Construct ***z*** and ***H***, and calculate ***e***:

(39) $$z_{t}={\left[z_{1,t}^{T},z_{2,t}^{T},\cdots ,z_{m,t}^{T}\right]}^{T},\;H_{t}={\left[H_{1,t}^{T},H_{2,t}^{T},\cdots ,H_{m,t}^{T}\right]}^{T},\;e_{t}=z_{t}-H_{t}x_{t|t-1}$$

Step 2: Calculate *d* and then update ***R***:

(40) $$d_{t}=\frac{1-b}{1-b^{t+1}},\;R_{t}=\left(1-d_{t}\right)R_{t-1}+d_{t}\left(e_{t}e_{t}^{T}-HP_{t|t-1}H_{t}^{T}\right)$$

Step 3: Update ***Y****_t_* and ***y****_t_*:

(41) $$Y_{t}=Y_{t|t-1}+H_{t}^{T}R_{t}^{-1}H_{t},\;y_{t}=y_{t|t-1}+H_{t}^{T}R_{t}^{-1}\left(e_{t}+H_{t}x_{t|t-1}\right)$$

Output phase: In this phase, *P_M_* and *T_M_* are extracted from ***y*** as outputs. First, ***y****_t_* are transformed back into ***x****_t_*. Subsequently, *P_M_* and *T_M_* are extracted through the output matrix (denoted as ***C***):

(42) $$x_{t}=Y_{t}^{-1}y_{t},\left[\begin{array}{c} P_{M}\\ T_{M} \end{array}\right]=Cx_{t}=\left[\begin{array}{ccc} 1 & 0 & 0\\ 0 & 1 & 0 \end{array}\right]x_{t}$$

At this point, the algorithm completes one full “prediction–update–output” cycle. Subsequently, the algorithm returns to the prediction stage, using ***x****_t_* and ***Y****_t_* as the initial conditions for the next time step to commence a new round of recursive calculation.

### 3.4. Sensor Calibration

Sensor measurement errors can propagate through the overall estimation chain and eventually manifest as altitude errors. Therefore, a calibration procedure needs to be performed to mitigate the impact of sensor errors on the proposed altimetry system. In practice, sensor errors can be simplified into two categories: offset (bias) errors and gain (scale) errors. Gain calibration typically requires dedicated metrology equipment and is difficult to carry out in field operations prior to UAV flights; hence, gain accuracy mainly depends on the sensor specifications. On the other hand, since the proposed system relies on synchronized differential measurements, the gain errors of the barometers tend to be largely common-mode between the ground reference station and the UAV-end unit; thus, they have a secondary impact on the resolved altitude. By contrast, offset errors are more sensitive to temperature and environmental conditions, but they can be compensated through a simple pre-flight calibration. Accordingly, this study focuses on offset calibration before flight. Taking the barometers as an example, the offset calibration procedure is described below.

Step 1: Co-located initialization. Place the ground reference station and the UAV (onboard unit) at the same location on the ground and keep them stationary for a short period until the pressure readings become stable. The altitude difference between the two units is assumed to be zero.

Step 2: Sampling and mean computation. Let the ground reference station contain *n* barometers and the UAV contain *m* barometers, with the total number *k* = *n* + *m*. Read the measurement of each barometer *P_i_* (optionally averaged over multiple samples to improve precision), and compute the mean value across all barometers:

(43) $$\bar{P}=\frac{1}{k}\sum_{i=1}^{k}P_{i}$$

Step 3: Offset estimation. Denote the calibration offset for the *i-th* barometer as *C_i_*, which is defined as the deviation of its reading from the mean:

(44) $$C_{i}=P_{i}-\bar{P}$$

Step 4: Offset compensation. After calibration, the compensated output of the *i-th* barometer is given as follows:

(45) $$P_{mi}=P_{i}-C_{i}$$

The compensated measurements *P_mi_* are then used as the pressure inputs for subsequent fusion and differential altimetry, effectively reducing the systematic errors caused by sensor offsets.

This study builds upon the IF by integrating the barometric altitude formulation, incorporating an efficient multi-sensor fusion scheme with adaptive mechanisms, and thereby establishing the AWA–AEIF algorithm tailored for UAV barometric altimetry.

## 4. Experiments and Results

This section describes the systematic validation of the proposed method conducted through numerical simulation, Hardware-In-the-Loop (HIL) simulation, and actual flight tests.

### 4.1. Simulation of Altitude Data Fusion Performance

#### 4.1.1. Static Performance

**Figure 7:**
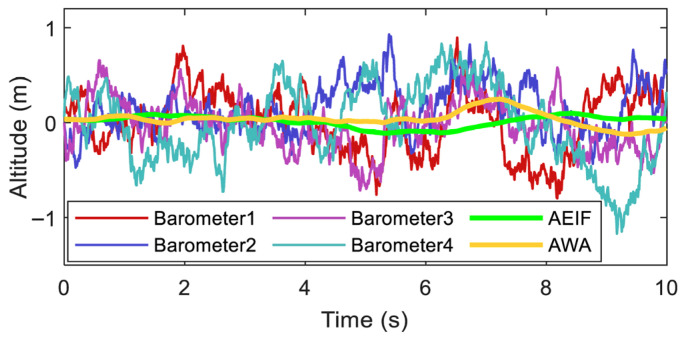
Noise suppression performance of AWA and AEIF algorithms under static conditions.

To evaluate the static fusion performance of the algorithm, a static altitude fusion simulation was conducted. The procedure was as follows: First, the outputs of four barometers were simulated, with their temperature and pressure values set to 20 °C and standard atmospheric pressure, respectively. On this basis, high-frequency noise (simulating measurement noise) and low-frequency noise (simulating zero drift) were superimposed onto the temperature–pressure data. Subsequently, the temperature and pressure were converted into altitude according to the pressure–height formula. Then, the AWA and AEIF algorithms were applied to fuse the four sets of temperature–pressure data. Finally, the fused temperature–pressure results were converted into altitude estimations, and the fusion performance was evaluated based on the altitude measurement noise. The simulation results are illustrated in Figure 7.

To quantitatively compare the precision of these methods, the Root Mean Square Error (RMSE) was used as a performance metric, which is defined as follows:

(46) $$RMSE=\sqrt{\frac{\sum_{n=1}^{N}{\left(h_{m,n}-h_{t,n}\right)}^{2}}{N}}$$

where *h_t,n_* and *h_m,n_* represent the ground truth and the measured value of altitude at the *n*-th sampling point, respectively, and *N* denotes the total number of samples. In various numerical simulations, the average of 10 simulation runs was taken as the final RMSE. In this simulation, *h_t_* was set to zero. The RMSE values for the six altitude curves illustrated in Figure 7 are summarized in Table 1.

The simulation results indicate that the raw sensor data exhibits severe fluctuations (with a maximum divergence of approximately 1 m), whereas the altitude curves fused by the AWA and AEIF algorithms are significantly smoother. The average RMSE of the four single-sensor altitude measurements is approximately 0.333 m; after fusion, the AWA and AEIF algorithms reduce the RMSE by approximately 70.3% and 68.5%, respectively.

#### 4.1.2. Dynamic Performance

**Figure 8:**
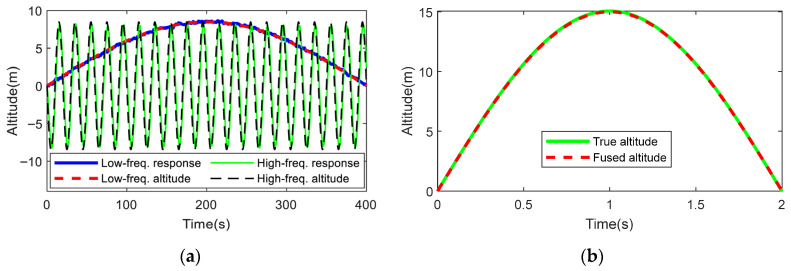
Comparison of dynamic altitude fusion results: (**a**) Frequency response of AWA; (**b**) Trajectory tracking by AEIF.

To evaluate the dynamic fusion performance of the algorithm, a dynamic altitude data fusion simulation was conducted. The procedure was as follows: First, low-frequency and high-frequency sinusoidal altitude curves were generated to simulate different motion patterns of the UAV. Subsequently, these altitude profiles were converted into temperature–pressure data, with noise superimposed onto the signals. Finally, the AWA and AEIF algorithms were applied to fuse the temperature–pressure data, which were then converted back into altitude curves. The simulation results are illustrated in Figure 8.

The simulation results indicate that the AWA algorithm effectively fuses slow-varying signals (period of 800 s, RMSE = 0.111 m). However, it exhibits a significant time lag when processing fast-varying signals (period of 20 s), with the RMSE increasing to 1.789 m, validating that its suitability is limited to slowly changing scenarios. Conversely, Figure 8b demonstrates that the AEIF algorithm achieves tight tracking even when the altitude variation rate reaches 15 m/s, maintaining a low RMSE of 0.078 m.

### 4.2. Simulation of Adaptive Performance

#### 4.2.1. Adaptive Standard Deviation Fusion

**Figure 9:**
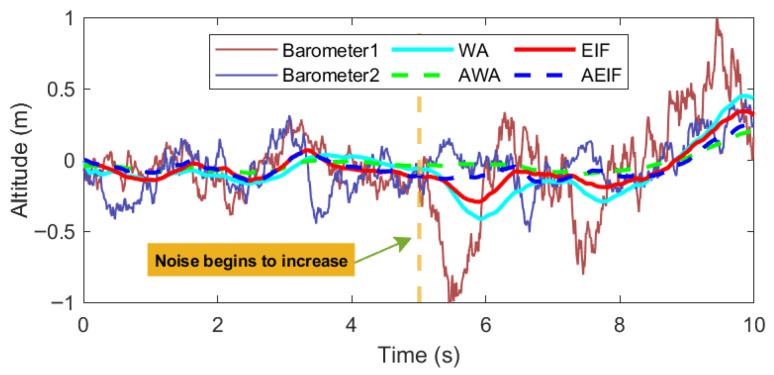
Comparison of adaptive and standard fusion results under varying noise.

To demonstrate the fusion effectiveness of adaptive standard deviation, the following simulation was performed: First, two sets of barometric data with identical standard deviations were generated. Subsequently, the standard deviation of the second half of one data set was doubled. Finally, the AWA, AEIF, WA (standard Weighted Average), and EIF algorithms were employed for fusion, and the results were converted into altitude values. The simulation results are illustrated in Figure 9, and the RMSE calculation results are summarized in Table 2.

The altitude curves show that during the 0–5 s interval (when noise characteristics remain constant), the estimation curves of all fusion algorithms almost overlap, with consistently low RMSE values. However, in the latter half from 5 to 10 s (after the noise of one data set increases), the curves begin to diverge. The algorithms with fixed standard deviations (WA and EIF) exhibit significant deviations from the raw data, with their RMSE surging by 112% and 167%, respectively. In contrast, the algorithms with adaptive standard deviations (AWA and AEIF) maintain superior tracking capabilities, with their RMSE increasing only slightly to 0.132 and 0.084 m, respectively. Overall, the RMSE of the adaptive algorithms is significantly lower than that of the fixed-standard-deviation algorithms.

#### 4.2.2. Estimation of Sensor Standard Deviation

**Figure 10:**
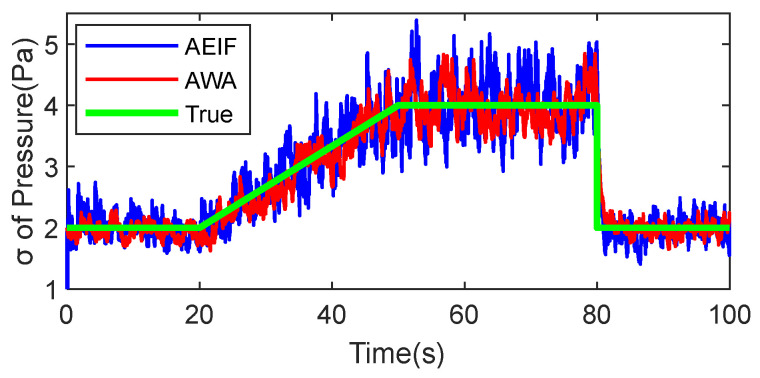
Estimation of dynamic barometric noise intensity using AWA and AEIF algorithms.

To evaluate the capabilities of the AWA and AEIF algorithms in estimating sensor standard deviation, a standard deviation estimation simulation was conducted using barometric data as a case study. The methodology was as follows: First, a set of barometric noise sequences with dynamic standard deviations was generated, encompassing three distinct phases: steady-state, slow-varying, and fast-varying. Subsequently, this set of noise sequences was superimposed onto standard atmospheric pressure to construct the simulated barometric data for estimation. Finally, the AWA and AEIF algorithms were applied to estimate the standard deviation of the noise. The simulation results are illustrated in Figure 10.

The data curves demonstrate that both the AWA and AEIF algorithms can effectively track variations in the standard deviation, with estimation errors of approximately ±0.8 and ±1.2 Pa, respectively. Collectively, both algorithms achieve relatively high-precision estimation of the time-varying standard deviation.

### 4.3. Simulation of Multi-Sensor Configurations

#### 4.3.1. Sensor Combinations

**Figure 11:**
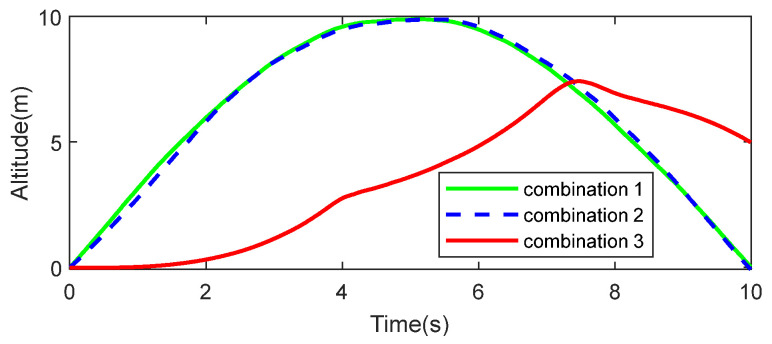
Performance comparison of various sensor combinations for dynamic altitude fusion.

To evaluate the contribution of the GPS and accelerometer to the altimetry precision, a comparative simulation of different sensor combinations was conducted. The configurations were as follows: Combination 1: barometer + GPS + accelerometer; Combination 2: barometer + accelerometer; Combination 3: barometer + GPS. In the simulation, the ground-truth altitude of the UAV was programmed to vary sinusoidally between 0 and 10 m. The simulation results are illustrated in Figure 11.

The simulation results show that the estimation curves of Combinations 1 and 2 are highly consistent, with RMSEs of 0.101 and 0.142 m, respectively. This indicates that the inclusion of the GPS leads to only a slight reduction in the RMSE, showing a marginal performance difference. In contrast, the tracking curve of Combination 3 exhibits significant lag and distortion, with the RMSE increasing sharply to 4.7 m. This demonstrates that the inertial information provided by the accelerometer is crucial for maintaining real-time tracking. By comparison, the contribution of the GPS primarily lies in further reducing the RMSE. Therefore, in GPS-denied environments (such as indoor flight), the combination of a barometer and an accelerometer can still achieve relatively high-precision altitude estimation.

#### 4.3.2. Sensor Number

To quantitatively explore the correlation between fusion precision (characterized by RMSE) and the number of barometers (denoted as *N*_b_), as well as between computational costs and *N*_b_, a parametric simulation experiment was performed. The computational cost (including complexity and memory usage), denoted as CC, is defined as the square of the state vector dimension and is calculated as follows:


(47) $$CC={\left[\dim(x)\right]}^{2}={\left(1+2N_{b}\right)}^{2}$$

**Figure 12:**
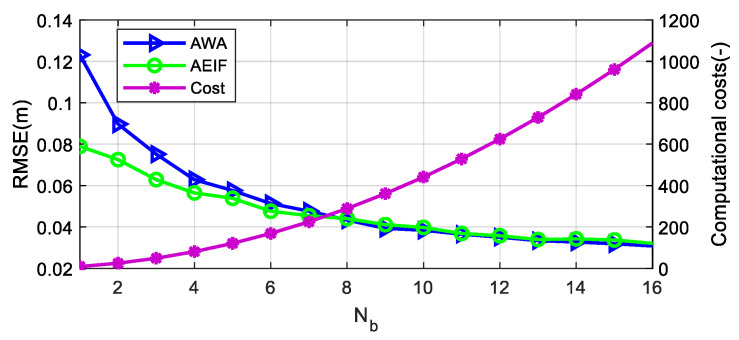
Correlation of RMSE and computational costs with the scaling of barometer quantity.

The methodology was as follows: First, *n* sets of temperature–pressure noise sequences with identical standard deviations were generated. Subsequently, the AWA and AEIF algorithms were applied to fuse the data, and then the RMSE and *CC* were calculated after converting the results into altitude profiles. Finally, the RMSE and CC were recalculated after changing *N*_b_. The simulation results are illustrated in Figure 12.

The test data demonstrate that for both the AWA and AEIF algorithms, the RMSE decreases approximately according to a logarithmic law as *n* increases. When *n* is small (e.g., *n* < 4), the RMSE of the AEIF algorithm is significantly lower than that of the AWA algorithm; however, as n increases (*n* > 8), the RMSE values of the two algorithms gradually converge. When *n* is greater than 8, *CC* increases rapidly as *n* increases. Therefore, from an engineering perspective, selecting a value between 4 and 8 for *n* yields the optimal cost-effectiveness, balancing measurement precision with system complexity.

### 4.4. Comparison of Altimetry Methods

#### 4.4.1. Numerical Simulation

**Figure 13:**
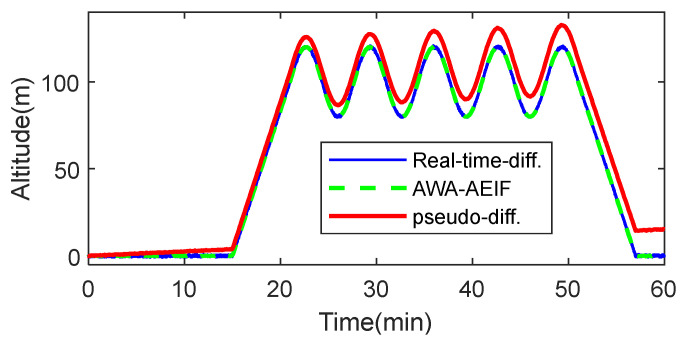
Mission-wide altitude tracking results for different differential and fusion methods.

To compare the performance of the three flight altitude measurement methods, namely pseudo-differential, real-time differential, and AWA–AEIF, the following simulation was conducted: First, the reference altitude profile for a UAV, encompassing four typical phases (standby, take-off, cruise, and landing), was generated. Subsequently, this altitude profile was converted into four corresponding sets of temperature–pressure profiles for the UAV-end unit, and four sets of ground-based reference data affected by atmospheric condition changes were also generated. Finally, the three methods were applied to resolve the altitude. The simulation results are depicted in Figure 13, and the RMSE calculation results are summarized in Table 3.

The simulation curves and data reveal that the pseudo-differential method fails to correct for time-varying atmospheric errors, resulting in measurement drift that accumulates over time. Its RMSE increases cumulatively from 2.45 m during standby to 13.82 m upon landing, with an overall RMSE of 9.05 m. Although the real-time differential method compensates for these errors using data from the ground-end reference station, it remains affected by the noise and drift of the barometer, with its stage-wise RMSE maintained between 0.274 and 0.296 m and an overall value of 0.288 m. The AWA–AEIF method further suppresses sensor noise, reducing the stage-wise RMSE to approximately 0.1 m, thereby achieving decimeter-level precision.

#### 4.4.2. HIL Simulation

**Figure 14:**
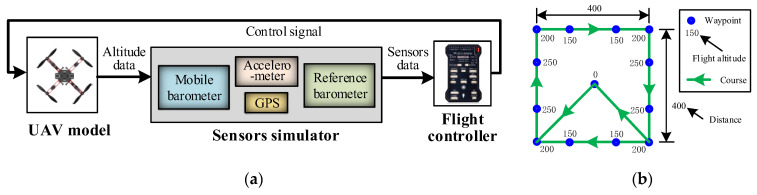
HITL simulation system: (**a**) Structural diagram; (**b**) Flight trajectory (in m).

To evaluate the performance of the proposed altitude measurement algorithm under conditions approximating real flight, a HIL simulation system was employed as the test platform. This platform utilizes real flight control hardware and a high-fidelity UAV dynamics model to construct a virtual flight environment. During the simulation, the true altitude provided by the virtual environment and the measured values output by the algorithm were acquired synchronously for comparison. The structure of the HIL system and the test flight path are illustrated in Figure 14.

**Figure 15:**
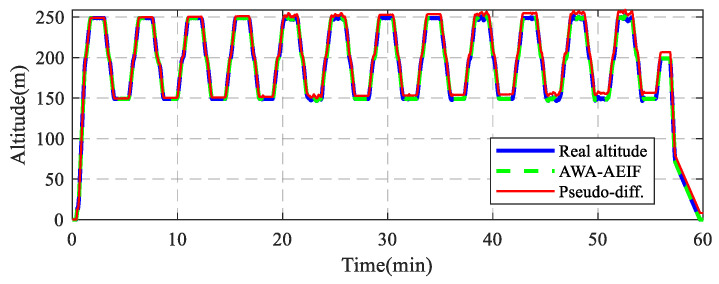
Comparative altitude tracking performance of pseudo-differential and AWA–AEIF.

The HIL simulation system consists of three main components—a UAV dynamics model, a sensor simulator, and a real flight controller—as illustrated in Figure 14a. During the simulation, the UAV model output the true altitude information of the UAV. Subsequently, the sensor simulator generated noisy sensor signals based on the altitude data, including ground-end temperature–pressure signals, mobile-end temperature–pressure signals, GPS velocity, and acceleration. These sensor data were transmitted in real time to the flight controller, where the flight altitude was estimated using both the pseudo-differential method and the AWA–AEIF method. The flight controller then output control signals, which were fed back to the UAV model, thereby achieving closed-loop flight control. The flight path configured for the simulation was as follows: The UAV took off from an altitude of 0 m and followed a square trajectory. The altitudes of the waypoints were sequentially set to 150, 200, 250, and 200 m, forming a periodic flight mission with altitude variations. To simulate atmospheric pressure perturbations encountered in real-world environments, a low-frequency time-varying offset was superimposed on the temperature–pressure data. The simulation results are shown in Figure 15.

The simulation results indicate that the flight altitude curve estimated using the AWA–AEIF method nearly completely overlaps with the true altitude curve, with a corresponding RMSE of only 0.1 m. In contrast, the altitude estimated by the pseudo-differential method continuously deviates from the true value over the entire flight duration, resulting in a corresponding RMSE of 4.3 m.

### 4.5. Physical Flight Tests

#### 4.5.1. Indoor Flight Test

**Figure 16:**
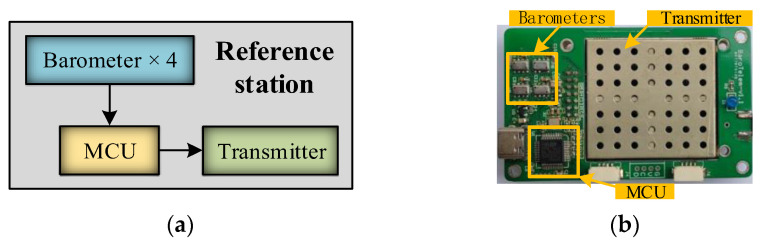
Barometric reference station: (**a**) Functional architecture; (**b**) Physical prototype.

Indoor flight represents one of the critical application scenarios for UAVs. To evaluate the practical performance of the AWA–AEIF method in indoor environments, an indoor flight test was conducted using a multi-rotor UAV platform. The test system consists of two primary components: a barometric reference station and the UAV platform. The architecture and the physical implementation of the barometric reference station are illustrated in Figure 16.

The barometric reference station consists of four barometers, a microcontroller unit (MCU), and a wireless transmitter. The selected barometer model is the MS5611 (TE Connectivity, Schaffhausen, Switzerland) [39], with a pressure resolution of approximately 4 Pa and a temperature resolution of approximately 0.01 °C. The MCU is a high-performance STM32F302 embedded processor (STMicroelectronics, Catania, Italy), responsible for data acquisition and AWA fusion calculations. The transmitter (SDR400, SINOSUN, Shenzhen, China) has a power output of 1 W, achieving a communication range of up to 10 km, and is used to transmit temperature–pressure data to the UAV.

**Figure 17:**
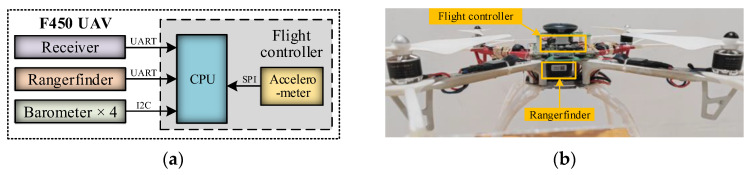
Indoor flight test platform: (**a**) Functional architecture; (**b**) F450 UAV.

An F450 quadrotor UAV was selected as the flight platform. Its structural schematic (highlighting the components relevant to altitude measurement) and the physical prototype are illustrated in Figure 17. The hardware configuration primarily includes a flight controller, four barometers (Model: MS5611, Interface: I2c), an accelerometer (Model: ICM20689, InvenSense, San Jose, CA, USA [40], Interface: SPI), a laser rangefinder, and a wireless receiver (SDR400, SINOSUN, Shenzhen, China). It should be noted that the GPS module was not utilized in this experiment, as the indoor test environment could not receive satellite signals. The flight controller is powered by a high-performance STM32H743 processor. A laser rangefinder (Model: TF02-Pro, Benewake, Beijing, Co., Ltd., Beijing, China [41], Interface: UART) with a measurement precision of 2 cm served as the altitude ground truth (reference benchmark). The receiver was paired with the transmitter of the ground reference station to acquire real-time temperature–pressure data from the base station, and the data transmission rate was 1 Hz. The performance specifications of the sensors employed in the experimental platform are summarized in Table 4.

In differential measurements, time misalignment between data sources may introduce additional errors. For differential barometric altimetry, however, the reference station’s temperature–pressure variations are typically slow. As shown in Figure 1, the reference altitude changes at approximately 7 m/h, which corresponds to less than 0.002 m/s. Therefore, even if the reference station’s data and the UAV-end measurements exhibit a time offset on the order of 1 s, the induced altitude error remains negligible. Based on this analysis, a data transmission rate of 1 Hz is sufficient to ensure accurate differential altitude estimation in our system.

**Figure 18:**
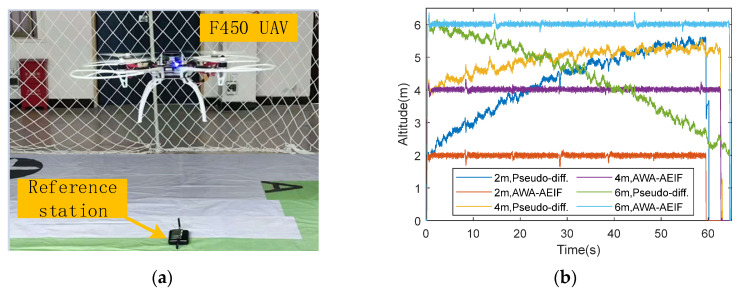
Indoor flight tests: (**a**) Experimental scenario; (**b**) Test results.

The test procedure was conducted as follows: The UAV followed a predefined flight path indoors. During the flight, altitude data measured by the laser rangefinder, the pseudo-differential method, and the AWA–AEIF method were recorded synchronously. To investigate the impact of atmospheric pressure variations at different times of day on the altimetry results, field tests were conducted during three separate time periods: morning (10:00–11:00), noon (13:00–14:00), and afternoon (17:00–18:00). The flight altitude was sequentially set to 2 m, 4 m, and 6 m for these periods, respectively. During the field tests, apart from the airflow disturbance induced by the UAV itself, no other significant external factors were present that could affect the altitude measurements. The test scenario and results are shown in Figure 18.

To analyze the measurement accuracy, the data from the laser rangefinder was taken as the ground truth, and the following RMSE formula was employed for quantitative analysis:

(48) $$RMSE=\sqrt{\frac{\sum_{n=1}^{N}{\left(h_{m,n}-h_{ref,n}\right)}^{2}}{N}}$$

where *h_ref,n_* denotes the ground-truth altitude measured by the laser rangefinder at the *n*-th sampling point, *h_m,n_* represents the corresponding estimated altitude, and *N* is the total number of samples. Given that the test duration was sufficiently long (>1 h), a single RMSE calculation was sufficient to reflect the error magnitude; therefore, only the single-run RMSE was computed. These quantitative results are summarized in Table 5.

The test results (Figure 18b and Table 5) show that across the three different time periods, the altitude measurements obtained using the pseudo-differential method were significantly affected by environmental pressure variations. In the morning, as the ambient pressure gradually increased, the measured altitudes were biased toward low readings, with a maximum error of +3.62 m. At noon, when the pressure changed moderately, the maximum error was +1.59 m. In the afternoon, with the ambient pressure gradually increasing, the measured altitudes were biased toward low readings, showing a maximum error of approximately −3.95 m, and the RMSE approached approximately 2 m. In contrast, the altitudes measured by the AWA–AEIF method remained stable around 2, 4, and 6 m in the morning, at noon, and in the afternoon, respectively, with an average RMSE of about 0.132 m.

#### 4.5.2. Outdoor Flight Test

**Figure 19:**
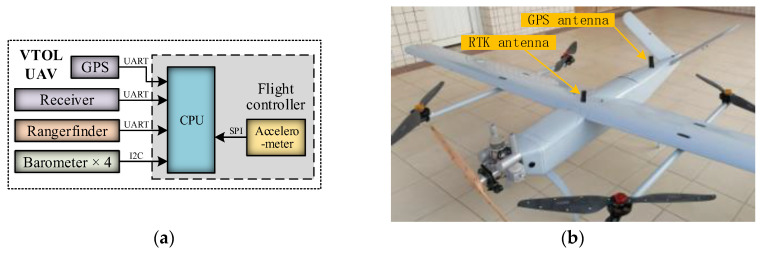
Outdoor flight test platform: (**a**) Functional architecture; (**b**) VTOL UAV.

Outdoor flight represents the primary application scenario for UAVs. Compared with indoor flight, outdoor flight typically operates at higher altitudes, where the effects of airflow disturbance on barometers are more pronounced. To evaluate the practical performance of the AWA–AEIF method under such complex conditions, a long-endurance fuel-powered Vertical Take-Off and Landing (VTOL) UAV was selected as the test platform. A schematic diagram and a photograph of the actual platform are shown in Figure 19.

The flight controller, barometers, accelerometer, and receiver of this outdoor UAV platform retain the same configuration as that of the indoor platform. The primary modifications involve the addition of a GPS module (NEO-M8N, u-blox, Thalwil, Switzerland) for measuring vertical velocity and the replacement of the original rangefinder with Real-Time Kinematic (RTK) technology. The RTK system (UM982, Unicore Communications, Inc., Beijing, China), serving as a high-precision positioning device with an altitude measurement error of less than 5 cm, was used to determine the UAV’s true flight altitude. Furthermore, to optimize signal reception performance, both the GPS and RTK antennas were mounted on top of the fuselage.

**Figure 20:**
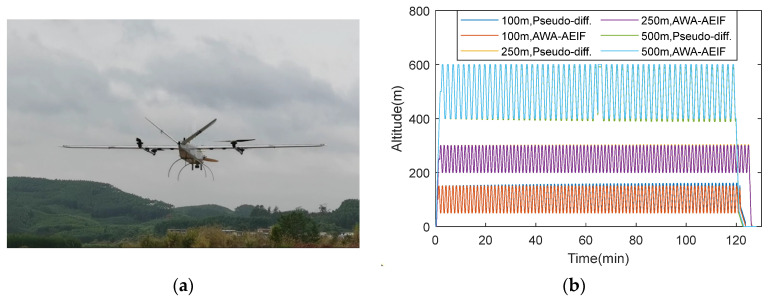
Outdoor flight tests: (**a**) Experimental scenario; (**b**) Test results.

During the outdoor flight tests, the UAV followed a predefined square trajectory three times, with the flight altitudes set at 100, 250, and 500 m. Dynamic altitude changes were introduced during the flight tests. The altitude measurements obtained using the RTK system, the pseudo-differential method, and the AWA–AEIF method were recorded synchronously. During the tests, the weather conditions were cloudy with intermittent gusts, with wind speeds ranging from approximately 0 to 4 m/s. The test scenario and results are presented in Figure 20.

The RMSE for each method was calculated based on Equation (48), where *h_ref_* represents the measurement value from the RTK system. The results are summarized in Table 6.

The data curves demonstrate that the altitudes measured by the AWA–AEIF method stably track the preset values across the three flight altitudes of 100, 250, and 500 m, exhibiting periodic variations that match the flight path. In contrast, the output curve of the pseudo-differential method gradually deviates from the preset values. The quantitative results indicate that the pseudo-differential altimetry method exhibits relatively large overall RMSE values (ranging from 1.82 to 4.76 m), with significant fluctuations across different altitudes and time periods. In contrast, the fusion-based altimetry method (AWA–AEIF) achieves smaller RMSE values (ranging from 0.31 to 0.94 m); however, its error gradually increases as the flight altitude rises from 100 to 500 m.

## 5. Discussion

This study investigates UAV flight altitude measurement based on barometric altimetry, with a focus on mitigating errors induced by atmospheric condition variations and sensor measurement inaccuracies, while improving dynamic performance, robustness, and adaptability.

To mitigate the impact of atmospheric condition variations, a measurement architecture based on a barometric reference station and a mobile station is proposed. Results from numerical simulations (Figure 13 and Table 3), HIL simulations (Figure 15), indoor flight tests (Figure 18b and Table 5), and outdoor flight tests (Figure 20b and Table 6) show that, with this architecture, altitude measurements become nearly insensitive to atmospheric variations. Compared with the pseudo-differential barometric altimetry method, the altitude measurement error is significantly reduced. It should be noted that introducing a barometric reference station increases equipment cost. Therefore, this solution is more suitable for industrial-grade UAVs with high performance requirements and relatively low cost sensitivity, whereas its applicability to consumer-grade UAVs may be limited.

To improve sensor measurement accuracy, a dual-layer fusion method is designed, employing AWA at the ground end and AEIF at the UAV end for barometric data fusion. Static fusion results from numerical simulations (Figure 7 and Table 1) show that the proposed method significantly reduces the measurement standard deviation, achieving a reduction of more than 70% relative to the raw measurements. Flight altitude measurement simulations (Figure 13 and Table 3) and HIL simulations (Figure 15) indicate an altitude RMSE of approximately 0.1 m. Indoor flight tests yield an RMSE of about 0.13 m, maintaining decimeter-level accuracy. In outdoor flight tests, relatively high accuracy is still achieved at low altitude (100 m), with an RMSE of 0.31 m. However, the RMSE increases with flight altitude. This increase can be attributed to two factors: (i) the inherent error of the pressure–height formula increases with altitude, and (ii) during high-altitude, high-speed flight, barometers are subject to stronger airflow disturbances, which degrade pressure measurement accuracy.

To enhance dynamic performance, robustness, and adaptability, a filtering model based on the pressure–height formula is designed to preserve filtering effectiveness while maintaining adequate dynamic response. Dynamic fusion simulations (Figure 8) and flight altitude measurement simulations (Figure 13 and Table 3) indicate that the RMSE does not increase substantially during rapid altitude changes. In addition, a sensor standard-deviation adaptive algorithm is designed to address cases where the sensor standard deviation is difficult to obtain accurately or varies over time. Adaptive standard deviation simulations (Figure 9 and Table 2) show a lower RMSE when the adaptive standard deviation is applied. The standard deviation estimation simulation (Figure 10) further indicates that the algorithm can estimate the sensor standard deviation with good accuracy. Sensor-combination fusion simulations (Figure 11) and indoor flight test results (Figure 18b and Table 5) show that a low RMSE can be maintained even without GPS-provided velocity information.

The performance improvements of the AWA–AEIF framework can be explained mechanistically. The AWA module suppresses low-frequency atmospheric drift at the reference station, stabilizing the differential baseline. The AEIF, built on a physics-consistent state model, enhances dynamic estimation by modeling the coupling among acceleration, velocity, altitude, and pressure. Meanwhile, adaptive noise estimation adjusts the measurement covariance in real time, improving robustness under time-varying disturbances. Together, these mechanisms reduce steady-state bias, enhance dynamic tracking performance, and lead to the consistent RMSE reductions observed in simulations, HIL tests, and real flights.

Regarding performance versus cost, Figure 12 shows that the RMSE reduction gradually saturates as the number of barometers increases, whereas computational cost increases rapidly. Therefore, an appropriate trade-off between performance improvement and implementation cost should be considered when selecting the number of sensors. Regarding real-time capability, the update rate of altitude estimation is typically low (approximately 20 Hz). Industrial-grade UAV flight controllers generally operate at high clock frequencies (>400 MHz) and are equipped with floating-point units. With this computational capability, the algorithm runtime remains on the order of milliseconds. Consequently, the proposed AWA–AEIF algorithm can satisfy real-time processing requirements.

A comparison is made between the barometric altimetry methods reported in Refs. [4,17,24] and the AWA–AEIF method proposed in this paper. The additional sensors, algorithms, and accuracy metrics used by each method are summarized in Table 7. Since these comparative methods use barometers only at the UAV end, they essentially belong to pseudo-differential barometric altimetry. Specifically, the dual-layer fusion method in Ref. [17] achieves an accuracy of approximately 0.1 m over a short duration (60 s), but does not report long-term stability or dynamic response. Ref. [24] employs a Kalman filter to integrate rangefinder data and achieves a static accuracy of 0.02 m, benefiting from the high precision of the rangefinder; however, the study does not discuss system performance during rangefinder outages caused by external factors, such as obstacles beneath the UAV. Ref. [4] mitigates barometric drift using a high-precision gyroscope combined with an NPLSSVM/KF framework, achieving decimeter-level accuracy, but the high cost and payload of such gyroscopes may limit their integration into standard UAV platforms. In contrast, the proposed method maintains decimeter-level accuracy during long-term operations. Although a barometric reference station is introduced, the required hardware (accelerometers, GPS, transmitters, and receivers) can be multiplexed with the UAV’s existing communication and navigation systems. Therefore, the incremental cost is mainly associated with adding multiple barometers. Overall, the proposed approach achieves a favorable balance between sensing performance and hardware expenditure.

## 6. Conclusions

To improve UAV altitude measurement accuracy, this paper proposes a reference–mobile station sensing architecture combined with a dual-layer fusion algorithm, denoted as AWA–AEIF. The theoretical feasibility of the proposed method is demonstrated through mathematical derivation and error analysis, and its effectiveness is validated through simulations and experimental tests. During long-duration altitude measurements, the RMSE values in the HIL simulation, indoor flight tests, and outdoor flight tests were approximately 0.1 m, 0.13 m, and 0.94 m (at an altitude of 500 m), respectively, achieving decimeter-level (indoor) and meter-level (outdoor) measurement accuracy. The accuracy improvements are achieved in the following three aspects:

1. The barometric reference station–mobile station architecture effectively mitigates the influence of environmental pressure variations, making the measurement error nearly time-invariant and substantially improving measurement stability.
2. The AWA–AEIF dual-layer information fusion algorithm suppresses sensor measurement noise and zero drift, further improving measurement precision and long-term stability.
3. The filtering model based on the pressure–height formula provides favorable dynamic performance, while the adaptive estimation algorithm for sensor standard deviation improves robustness and adaptability.

The proposed altimetry method requires a specific hardware architecture, which introduces a modest increase in equipment cost (approximately $10). Therefore, it is more suitable for UAV platforms with higher performance requirements. From a deployment perspective, the proposed AWA–AEIF framework is compatible with typical UAV constraints in terms of onboard computation and communication. The differential sensing architecture and adaptive noise modeling reduce sensitivity to slow atmospheric variations, although performance may degrade under highly unstable atmospheric conditions, which is a common limitation of barometric altimetry. In addition, the proposed framework can be extended to multi-platform sensing networks in which multiple UAVs share reference stations or exchange pressure information; this will be explored in future work.

## Figures and Tables

**Table 1:** RMSE comparison of altitude estimation methods under static conditions (in m).

	Barometer 1	Barometer 2	Barometer 3	Barometer 4	AWA	AEIF
RMSE	0.344	0.349	0.314	0.323	0.099	0.105

**Table 2:** RMSE comparison between adaptive and standard fusion algorithms (in m).

Time	WA	AWA	EIF	AEIF
0–5 s	0.094	0.079	0.102	0.06
5–10 s	0.199	0.132	0.272	0.084
0–10 s	0.155	0.109	0.206	0.073

**Table 3:** RMSE comparison of altimetry methods across various flight mission phases (in m).

Method	Standby	Takeoff	Cruise	Landing	Whole Course
Pseudo-difference	2.45	4.6	9.4	13.82	9.05
Real-time-difference	0.274	0.296	0.295	0.289	0.288
AWA–AEIF	0.102	0.107	0.109	0.1.07	0.106

**Table 4:** Performance specifications of the primary sensors used in the experimental platform.

Sensors	Model	Data Rate(Hz)	Resolution	Accuracy
Barometer	MS5611	256–4096	0.065–0.012 mbar	−2.5–+2.5 mbar
Thermometer	MS5611	256–4096	<0.01 °C	−4.0–+4.0 °C
Accelerometer	ICM20689	5–4000	Noise Spectral Density: 150 µg/√Hz	−80–+80 mg
Rangefinder	TF02-Pro	1–10,000	1 cm	Repeatability: <2 cm @ 1σ

**Table 5:** The errors of altitude measurements in indoor flight tests (values are reported as RMSE/maximum error, in m).

Method	2 m, 10:00–11:00	4 m, 13:00–14:00	6 m, 17:00–18:00
Pseudo-difference	1.83/3.62	0.65/1.59	1.92/−3.95
AWA–AEIF	0.133/0.197	0.126/0.226	0.138/−0.254

**Table 6:** The errors of altitude measurements in outdoor flight tests (values are reported as RMSE/maximum absolute error, in m).

Method	100 m, 9:00–11:00	250 m, 12:00–14:00	500 m, 16:00–18:00
Pseudo-difference	4.05/10.24	1.82/4.55	4.76/−11.28
AWA–AEIF	0.31/0.78	0.65/0.92	0.94/−1.34

**Table 7:** Comparative analysis of representative altitude measurement methods and the proposed algorithm.

Source	Additional Sensors	Algorithm	Accuracy
Huang [17]	accelerometer, GNSS, radio altimeter	Dual-layer information fusion	0.096 m (short term)
Yu [24]	accelerometer, rangefinder	KF	<0.02 m (static)
Zhao [4]	RINS	NPLSSVM/KF	Decimeter-level
Ours	accelerometer, GPS (optional)	AWA–AEIF	0.10 m (simulation), 0.13 m (indoor), 0.31 m (outdoor 100 m)

## Data Availability

The original contributions presented in this study are included in the article material, and any further inquiries can be directed to the corresponding authors.

## Appendix A

This appendix presents the detailed derivation for the standard deviation of altitude measurement (*σ_h_*) discussed in Section 2.3.

To quantify the uncertainty of the real-time differential barometric altimetry results, the standard deviation of the altitude measurement, denoted as σ*_h_*, is employed as the evaluation metric. Provided that the standard deviations *σ_mi_* of all input quantities *m_i_* (namely, *T_R_*, *P_R_*, *T_M_*, *P_M_*) are known, σ*_h_* can be calculated according to the law of error propagation. Considering the potential correlations among the input quantities, the general calculation formula is given by [42]:

(A1) $$\sigma_{h}=\sqrt{\sum_{i=1}^{n}{\left(\frac{\partial M}{\partial m_{i}}\right)}^{2}\sigma_{m_{i}}^{2}+2\sum_{i=1}^{n}\sum_{j=i+1}^{n}\left(\frac{\partial M}{\partial m_{i}}\right)\left(\frac{\partial M}{\partial m_{j}}\right)Cov(m_{i},m_{j})}$$

where $Cov(m_{i},m_{j})=E\left[\left(m_{i}-E\left[m_{i}\right]\right)\left(m_{j}-E\left[m_{j}\right]\right)\right]$ denotes the covariance matrix, and *E*[·] represents the mathematical expectation. If the measurement errors of the input variables are uncorrelated, the corresponding covariances become zero, and Equation (A1) can be simplified as [42,43]:

(A2) $$\sigma_{h}=\sqrt{\sum_{i=1}^{n}{\left(\frac{\partial M}{\partial m_{i}}\right)}^{2}\sigma_{m_{i}}^{2}}$$

For the barometric formula, the altitude (*h*) is defined as a function *M*(*T_R_*, *P_R_*, *T_M_*, *P_M_*) following Equation (3):

$$h=7995.4\left(1+\frac{T_{R}+T_{M}}{546.3}\right)\ln\frac{P_{R}}{P_{M}}$$

To obtain the specific expression for σ*_h_*, the partial derivatives of *h* with respect to each input variable must be computed:

1. Partial derivative with respect to *T_R_*:

(A3) $$\frac{\partial h}{\partial T_{R}}=\frac{7995.4}{546.3}\ln\frac{P_{R}}{P_{M}}\approx 14.63\ln\frac{P_{R}}{P_{M}}$$

2. Partial derivative with respect to *T_M_*:

(A4)
$$\frac{\partial h}{\partial T_{M}}=\frac{7995.4}{546.3}\ln\frac{P_{R}}{P_{M}}\approx 14.63\ln\frac{P_{R}}{P_{M}}$$

3. Partial derivative with respect to *P_R_*:

(A5)
$$\frac{\partial h}{\partial P_{R}}=7995.4\left(1+\frac{T_{R}+T_{M}}{546.3}\right)\frac{1}{P_{R}}$$

4. Partial derivative with respect to *P_M_*:

(A6)
$$\frac{\partial h}{\partial P_{M}}=-7995.4\left(1+\frac{T_{R}+T_{M}}{546.3}\right)\frac{1}{P_{M}}$$

By substituting the partial derivatives derived from the barometric formula into Equation (A2), we obtain the final analytical expression for *σ_h_*:

(A7) $$\sigma_{h}=\sqrt{214.04{\left(\ln\frac{P_{R}}{P_{M}}\right)}^{2}\left(\sigma_{T_{R}}^{2}+\sigma_{T_{M}}^{2}\right)+{\left(7995.4+14.63\left(T_{R}+T_{M}\right)\right)}^{2}\left(\frac{\sigma_{P_{M}}^{2}}{P_{M}^{2}}+\frac{\sigma_{P_{R}}^{2}}{P_{R}^{2}}\right)}$$
